# Soil microbial improvement using enriched vinasse as a new abundant waste

**DOI:** 10.1038/s41598-023-49401-w

**Published:** 2023-12-14

**Authors:** Tahereh Kariminia, Mohammad A. Rowshanzamir, S. Mahdi Abtahi, Sabihe Soleimanian-Zad, Hamid Mortazavi Bak, Alireza Baghbanan

**Affiliations:** 1 Department of Civil Engineering, Isfahan University of Technology, Isfahan, 84156-83111 Iran; 2 Department of Food Science and Technology, College of Agriculture, Isfahan University of Technology, Isfahan, 84156-83111 Iran; 3 Research Institute for Biotechnology and Bioengineering, Isfahan University of Technology, Isfahan, 84156-83111 Iran; 4 School of Civil and Environmental Engineering, University of New South Wales, Sydney, 2052 Australia; 5 Department of Mining Engineering, Isfahan University of Technology, Isfahan, 84156-83111 Iran

**Keywords:** Biological techniques, Microbiology, Biogeochemistry, Engineering

## Abstract

This study proposes the use of vinasse, an inexpensive and readily available waste biopolymer, as a fundamental component of a waste culture medium that can enhance the effectiveness and cost-efficiency of the microbial-induced calcite precipitation (MICP) method for sustainable soil improvement. Vinasse enriched with urea, sodium caseinate, or whey protein concentrate is employed to optimize bacterial growth and urease activity of *Sporosarcina pasteurii (S. pasteurii)* bacterium. The best culture medium is analyzed using Taguchi design of experiments (TDOE) and statistical analysis, considering the concentration of vinasse and urea as effective parameters during growth time. To test the best culture medium for bio-treated soil, direct shear tests were performed on loose and bio-treated sand. The results demonstrate a substantial cost reduction from $0.455 to $0.005 per liter when using the new culture medium (vinasse and urea) compared to the conventional Nutrient Broth (NB) culture medium. Additionally, the new medium enhances soil shear strength, increasing the friction angle by 2.5 degrees and cohesion to 20.7 kPa compared to the conventional medium. Furthermore, the recycling of vinasse as a waste product can promote the progress of a circular economy and reduce environmental pollution. As ground improvement is essential for many construction projects, especially those that require high shear strength or are built on loose soil, this study provides a promising approach to achieving cost-effective and sustainable soil microbial improvement using enriched vinasse.

## Introduction

In the realm of soil stabilization, traditional methods encompassing chemical, petroleum-based, polymer-based, and biopolymer enhancements have found extensive application in fortifying soil strength properties and addressing the challenges posed by loose and desert soils. Nevertheless, these established approaches often present drawbacks, including high costs, significant demands on human and material resources, and the environmental concerns due to contamination, resource intensity, greenhouse gas emissions, and habitat disruption^1–3^.

In contrast, soil microbial improvement offers a potential solution without these disadvantages, providing increased soil strength and resilience against diverse environmental conditions, including freezing and melting cycles, as well as alternating wet and dry periods^4–7^. Hence, in recent decades, bio-cementation techniques have emerged as eco-friendly alternatives to traditional loose soil improvement methods^8–12^.

In this context, the microbial-induced calcite precipitation (MICP) method has emerged as a sustainable approach to soil improvement, aligning with sustainability goals by relying on natural processes, reducing resource use and energy footprint, mitigating soil erosion, and promoting long-term soil stability, thereby minimizing environmental harm and contributing to reduced environmental impact in soil improvement practices.

The process involves the fracturing of urea by bacterial urease enzymes (i.e. *Sporosarcina pasteurii (S. pasteurii)*, *Bacillus licheniformis*,* Bacillus megaterium*,* Bacillus thuringiensis*,* Pseudomonas sp*, and *Lysinibacillus sphaericus*), which then leads to the precipitation of calcite crystals between soil particles in the presence of calcium ions^13,14^. The detailed chemical reactions of the MICP process are presented in Eqs. (1) to (4):

1 $${{(NH}_{2})}_{2}CO+{H}_{2}O\stackrel{\mathrm{urease bacteria }}{\to }2N{H}_{3}+{CO}_{2},$$

2 $${NH}_{3}+{H}_{2}O\to {NH}_{4}^{+}+{OH}^{-},$$

3 $${CO}_{2}+{OH}^{-}\to {HCO}_{3}^{-},$$

4 $${Ca}^{2+}+{HCO}_{3}^{-}+{OH}^{-}\to {H}_{2}O+{CaCO}_{3}.$$

This method has demonstrated significant potential in various geotechnical applications, including enhancing soil strength^11,15–18^, mitigating liquefaction potential^19^, enhancing shear strength parameters of soil-steel interfaces^20^, repairing cracks^21–23^, reducing dust generation^24,25^, reducing efflorescence rate^26^, improving erosion resistance in diverse environmental conditions^27–30^, and the removal of heavy metals^31^.

Previous studies have shown that *Sporosarcina pasteurii (S. pasteurii)* bacterium enhances the efficiency of the MICP method considering the following reasons: being highly capable to hydrolyze urea, being nonpathogenic, being insensible to the contamination of culture media, and being possible to maintain this kind of bacterium for a long period of time^16,32–34^.

Studies have shown that the effectiveness of soil treatment using the MICP method is greatly influenced by the concentration of bacteria and the amount of their urease activity^35,36^. Accordingly, the optimal bacterial concentration for achieving ideal efficiency in the MICP method has been determined to be between 8 and 12 log (cfu/mL)^37,38^.

Although the biotechnology has demonstrated promising results at the laboratory scale, a major obstacle for its field-scale application is the high cost of nutrient culture media needed for cultivating the appropriate bacteria^18,39^.

The cost of implementing the technique commercially can be substantial due to the use of conventional growth media such as Nutrient Broth (NB), Tryptic Soy Broth (TSB), Luria Broth (LB), and others^40^, and studies have shown that it can account for up to 60% of the total cost of implementing the MICP method in the field^34,41^.

To address this challenge, alternative nutritional sources or protein-rich nutrients have been investigated for cultivating urease bacteria in MICP applications. For example, industrial or agricultural by-products such as black strap molasses, corn steep liquor, and agricultural activated sludges have been explored as potential alternatives to conventional growth media, as they can increase bacterial population and urease activity^42–47^.

In fact, besides the potential for low-cost bacterial culturing, nutritional waste materials are often discarded in large volumes. Therefore, it is important to explore more cost-effective bacterial culture media using available abundant waste.

**Figure 1:**
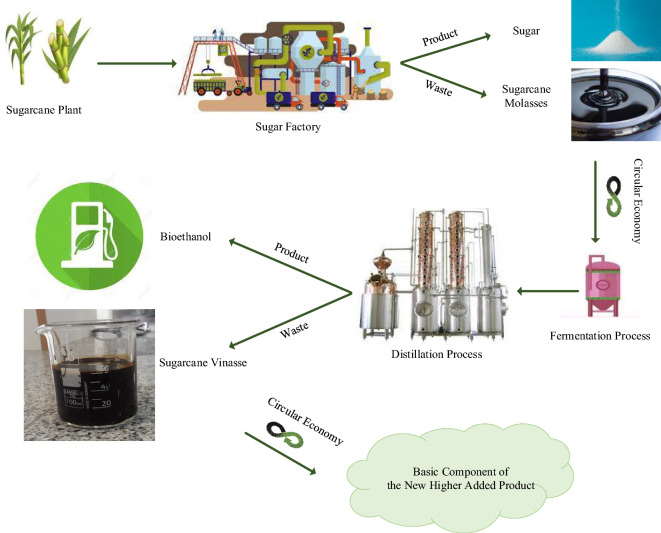
Potential circular bio-economy product of vinasse.

In the light of the above, one potential nutritional waste is the use of vinasse, a by-product of bio-ethanol production from sugarcane agricultural waste (Fig. 1). Vinasse contains high amounts of phosphorus, magnesium, calcium, amino acids, glucose, and a range of other micronutrients and macronutrients, as well as organic matter. This may make it an excellent candidate for use as a *S. pasteurii* culture medium in soil stabilization processes, and a potential circular economy solution^48–51^.

The production of one liter of bio-ethanol results in the generation of 10–18 L of vinasse, and the disposal of this waste in soil and water with high levels of concentration has led to significant environmental problems^50,52,53^. The disposal of a large volume of waste material, such as concentrated vinasse, can result in detrimental environmental effects due to factors including soil and water contamination, altered nutrient dynamics, increased Biochemical Oxygen Demand (BOD) leading to reduced oxygen levels in aquatic ecosystems, the potential release of alkaline compounds, and disruption of local ecosystems. These factors collectively contribute to ecological imbalances and degradation of environmental quality^54,55^.

Vinasse as a nutrient-rich waste material that is cost-effective and can be recycled, can also be utilized as a soil fertilizer at low concentrations due to its high nutrient content^56^. Previous studies have confirmed the positive effects of vinasse on soil physicochemical properties^57–60^. Within the realm of utilizing vinasse for soil enhancement, as far as the author is aware, no studies have been conducted to assess the influence of vinasse on soil strength parameters. Nonetheless, some research has delved into its impact on controlling runoff and mitigating soil erosion^61,62^, and Nikseresht et al.^63^ conducted wind tunnel experiments to assess the effectiveness of molasse and vinasse waste materials as microbial growth substrates for MICP in preventing soil loss under varying wind speeds. It was observed that both molasse and vinasse demonstrated substantial penetration resistance in dry conditions but raised concerns about durability in wet conditions. This increased resistance to wind erosion was attributed to their high calcium carbonate content, which remained unaffected by additional modifications, including the introduction of bacteria (e.g. *S. pasteurii*) and MICP cementing agents (e.g. CaCl_2_ and urea)^63^. However, their study did not address the aspects of *S. pasteurii* bacterial growth or its urease activity when utilizing enriched vinasse with essential protein supplements. These aspects are pivotal for comprehending the potential of these waste materials as a culture medium for *S. pasteurii* bacteria and assessing their impact on MICP-treated soil.

Therefore, the main objective of this study is to investigate the cultivation of the *S. pasteurii* bacterium and its urease activity using enriched vinasse with different supplements, as well as to examine the efficiency of the culture medium in treating MICP-treated soil.

To explain more, to reduce the primary costs of the MICP method, this study examines the capability of diluted vinasse by-product as the basic ingredient of culture media. In this context, *S. pasteurii* bacteria are cultured on enriched diluted vinasse with one of other supplements including fertilizer urea (U), sodium caseinate (NaCAS) or whey protein concentrate (WPC) which provide the necessary protein or nitrogen for bacterial growth. The NB + U medium is used as a reference conventional medium to examine bacterial growth, as it is known to be the cheapest among other conventional media^64^.

The combination of vinasse and fertilizer urea is found as the most efficient culture medium based on considering bacterial growth curves, urease activity, and the cost of each medium. The percentages of vinasse and fertilizer urea are optimized using TDOE, ANOVA, and ANOM methods. The bacteria cultured in the optimal medium are then used in the MICP method, and its effectiveness on soil strength enhancement is assessed through direct shear tests as well as SEM (Scanning Electron Microscope) images, and XRD (X-Ray Diffraction) analyses.

In the light on the above, the objective of this research is to enhance the cost-effectiveness and the efficiency of the MICP method for soil microbial improvement by introducing vinasse waste biopolymer as a valuable nutrient source for the *S. pasteurii* bacterium. This lowers construction expenses, encourages responsible waste management, strengthens infrastructure resilience, and aligns with sustainability objectives. In conclusion, this research has the potential to enhance the sustainability, cost-efficiency, and eco-friendliness of green soil stabilization projects, while also aiding large-scale desertification control efforts to reduce dust storms.

## Materials and methods

### Bacterial strain and culture media

To activate lyophilized ampoules of *S. pasteurii* (ATCC 11,859), the following steps were taken: Ensuring sterility, preparing a suitable culture medium with NB (3 g/L) + U (20 g/L) which consists of nutrient broth (NB: Merck, Germany) and urea fertilizer (U: Kermanshah Petrochemical Industries co., Iran), opening the ampoule under sterile conditions within a laminar hood, transferring the lyophilized material into the culture medium, incubating at 30 °C, monitoring bacterial growth, and subculturing when necessary.

Previous studies have indicated that 3 g/L of nutrient broth (NB) and 20 g/L of urea are required for optimal growth of *S. pasteurii* in soil microbial improvement applications^65,66^. To introduce a new culture medium, it is important to ensure that the new culture medium contains a similar amount of protein per liter of bacterial solution as the current culture medium. Nutrient broth contains approximately 1.4 g/L of protein when used at a concentration of 3 g/L. In contrast, the protein content of the used vinasse is about 20.1 g/L. To achieve the consistent protein content, it is necessary to dissolve approximately 70 mL of vinasse per liter of distilled water.

It is important to highlight that in this study, we utilized concentrated vinasse sourced from Sepahan Bio Product Co in Iran. This vinasse boasts significant quantities of various components, including phosphorus (610 mg/Kg), magnesium (656 mg/Kg), calcium (1.8%), amino acids such as glutamic acid, alanine, methionine, leucine, proline, and serine (ranging from 0.1 to 0.2%), glucose (2.3%), and a wide array of other micronutrients like iron (285 mg/Kg), zinc (18 mg/Kg), manganese (17 mg/Kg), and copper (4.5 mg/Kg). Additionally, it contains essential macronutrients like nitrogen (1.7%), sodium (1.6%), and potassium (3.3%), along with organic matter such as sugar (2.3%) and protein (20.1 g/L).

Furthermore, in order to cultivate *S. pasteurii* bacterium, it is necessary to enrich the NB with urea (i.e. 20 g of urea per liter) as a supplement source of nitrogen (N))^24,42^. In this study, new culture media consisting of vinasse (V) were enriched separately with three different protein sources: fertilizer urea (U), sodium caseinate (NaCAS), and whey protein concentrate (WPC). The selection of these supplements was based on the uncertain behavior of *S. pasteurii* bacterium in the combined media.

The selection of vinasse and protein sources (urea, sodium caseinate, whey protein concentrate) for the new culture media is driven by various factors supported by prior research^47,67,68^. Vinasse, rich in organic matter and nutrients, serves as an excellent carbon source, but its direct use is limited due to salinity and dense organic matter, which can be improved by combining it with other waste materials^69,70^. However, vinasse lacks sufficient protein for *S. pasteurii* growth, necessitating protein supplements. Urea is chosen based on studies demonstrating its ability to sustain urease-producing bacteria growth^24,42,65,66^. Sodium caseinate is considered due to previous research combining it with sugarcane molasses for *S. pasteurii*^71^. Whey protein concentrate is used to address environmental issues related to whey disposal^72,73^ and its potential impact on *S. pasteurii* behavior when added to vinasse-enriched culture media, as previous studies indicated some positive impact on urease activity^68^.

With fertilizer urea as a reference, which contains 46% nitrogen, the Kjeldahl conversion method was employed to assess the protein content of NaCAS and WPC. The goal was to determine the quantities of NaCAS and WPC required to match the protein content of 20 g/L urea, as determined by Kjeldahl (N × 6.38; where N represents the nitrogen percentage). This calculation yielded an equivalent of 58.7 g of protein (20 g of urea × 0.46 × 6.38). Subsequently, this value was divided by the respective protein percentages of NaCAS (91%) and WPC (70%) to ascertain the necessary amounts of these materials. Therefore, 64.5 g/L NaCAS with 91% protein content^74^, and 83.85 g/L WPC with 70% protein content^75^, were added to vinasse to introduce the culture media.

The performance of *S. pasteurii* bacteria was evaluated and compared among aforementioned culture media using growth curves and urease activity.

It should be emphasized that the term "urea" used in the current study's investigations specifically pertains to industrial-grade fertilizer urea.

### Growth curves and urease activity

Prior to bacterial cultivation, the culture media were autoclaved at 121 °C for 15 min and their pH was adjusted to 7^17,65,76^. It should be noted that when using culture media containing urea, the addition of urea should be sterilized through filtration under completely sterile conditions to prevent hydrolysis of the urea due to high temperatures. Following the sub-culturing of the bacteria into NB + U culture medium, the bacteria were inoculated into the new media and conventional medium.

In this study, to generate bacterial growth curves, the initial optical density (OD), representing biomass at a 600 nm wavelength, was set to 0.05 using a spectrophotometer device, with NB + U culture medium serving as a blank. Subsequently, the biomass was harvested at 4000rpm and 25 ± 2 ℃ for 15 min. The collected biomass was then introduced into the culture media contaminated with *S. pasteurii* bacteria and incubated in a shaker incubator at 30 °C and 130 rpm. Throughout the bacterial growth process (where time served as one of the parameters under investigation), the extent of growth was determined at various time points by enumerating the number of bacterial colonies, expressed as colony-forming units per mL (cfu/mL) (See Fig. S1 in the supplementary material for a schematic representation of using the Miles-Misra method to present The bacterial growth process)^77^.

Emphasizing the precision and commonality of bacterial colonies count in constructing growth curves, this study utilized the specialized Miles-Misra method to enumerate bacterial colonies. This method involves diluting the sample, spreading it on an agar plate, and allowing individual bacterial colonies to grow and become visible, enabling an accurate count of bacterial numbers^77^.

The urease activity of the *S. pasteurii* bacterium was assessed in the various culture media, including N + U, V + NaCAS, V + WPC, and V + U, using the Nessler method by using Nessler’s reagent. This method involves measuring the amount of ammonia released from hydrolyzed urea in different considered growth times, and the measurements were conducted at specific conditions: using a spectrophotometer device at wavelength of 425 nm, temperature at 25 °C, and a pH of 7^14,34^. The unit of urease activity is U/min indicating the amount of urease enzyme contained in 1 mL of culture media which can hydrolyze one micromole of urea per minute^39,78^.

Urease activity data played a crucial role in determining the optimal culture medium for MICP. The data were collected to measure how effectively *S. pasteurii* bacteria in different culture media converted urea into ammonia and bicarbonate ions, a vital step in MICP. By comparing urease activity across the various culture media, the culture medium that supported the highest urease activity, was selected as the optimal one for MICP. Accordingly, the results of the urease activity presented in Results and discussions section will be used to determine the optimum culture medium.

### Soil specifications

**Figure 2:**
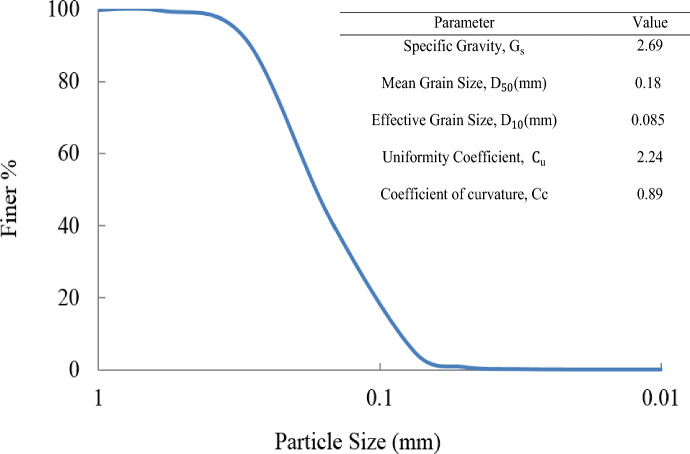
Grain size distribution and the main parameters of sand.

To evaluate the effectiveness of the MICP method, a series of direct shear tests were conducted on sandy soil (from Varzaneh desert margin at the central part of Iran) samples treated with the best combination culture media. Figure 2 displays the gradation curve and other specifications of the poorly graded sand.

### Preparation of cementation and bacterial solution

Urea and CaCl_2_ are the main reactants of the MICP process. In the process, urea serves as the carbon source for microbes, enabling the production of carbonate ions, which react with calcium ions from calcium chloride to form calcium carbonate, cementing soil particles together. According to the bio-cementation equations, the same molarities of urea (MW 60.06 g/mol) and calcium chloride (MW 110.98 g/mol) were required to produce one mole of CaCO_3_ precipitation^11,17,79^. Thus, the concentration of urea and CaCl_2_ is one mol/L in this study. To prepare the cementation solution, dissolved urea and calcium chloride in separate containers, then mixed them at the same ratio.

To examine the impact of the new optimal culture medium on the microbial improvement of sandy soil, polluted culture media at the stationary phase were harvested (4000 rpm at 25 ± 2 ℃, 15 min). The bacterial mass was washed, and fresh culture medium was utilized as a bacterial solution to nourish the bacteria in the soil medium. The pH of the bacterial solution was set at 7. To adjust the bacterial solution’s pH to 7, 2.12 g/L sodium bicarbonate (NaHCO_3_) as a pH buffer was used to create optimal conditions for MICP.

Sodium bicarbonate helps counteract the alkaline effects resulting from the production of carbonate ions during MICP, ensuring that the pH remains within the suitable range for calcium carbonate precipitation.

The conventional culture medium (NB (3 g/L) + U (20 g/L)) was utilized as the control culture medium in subsequent experiments.

### Soil samples preparation

**Figure 3:**
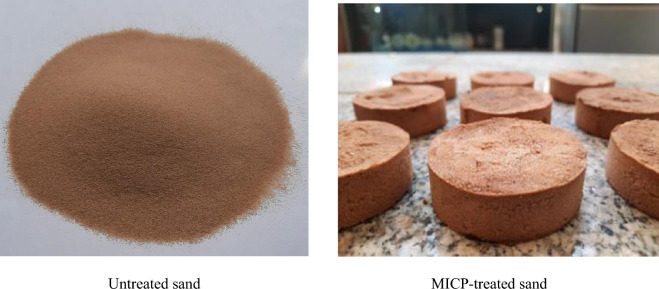
Sandy soil specimens: untreated vs. MICP-treated.

To remove any fine fraction, the sandy soil was initially washed with distilled water on a No. 200 mesh sieve and dried in an oven at 100 °C for 24 h. The bacterial solution was subsequently added to the soil sample prepared at relative density of 35% within a proper mold, a cylindrical mold with a diameter of 63 mm and a height of 20 mm, for conducting direct shear test after curing. The volume of the bacterial solution added was equal to the void volume of the soil^80^. In this study, void volume was 25.5 mL. The sample was allowed to rest for 1 h (fixation step), after which a volume of cementation solution equal to the void volume of the soil was injected into the samples at a rate of 0.35 L/h using a peristaltic pressure pump^14^. After 24 h, the cementation solution was injected into the samples again, but this time with a volume equal to half of the first cycle. Therefore, the total volume of the applied cementation solution was 38.25 mL. The samples were then left to cure for 10 days, to provide sufficient time for the bacterial and cementation processes to take place and strengthen the soil structure, at a temperature of 25 ± 2 ℃, which is considered the optimal temperature range according to previous studies^79,81–83^. Following the curing period, the stabilized soil samples were rinsed with distilled water. This step was taken to eliminate any soluble residues that may have remained due to the presence of the culture media in the soil. Finally, the samples were oven-dried for 24 h before conducting direct shear tests to eliminate any effects of suction on the results^16,20^. In this study, Fig. 3 exhibits images of both untreated soil and soil treated with MICP.

Both untreated soil and MICP-treated soil were subjected to direct shear tests as per ASTM D3080. To further examine the capability of new culture medium to precipitate the carbonate crystals among and on the surface of the soil particles, a series of SEM (Scanning Electron Microscopy) images and XRD (X-ray Diffraction) analyses were made on the treated samples.

### Experimental design and statistical analyses

After conducting a series of initial tests, the most appropriate supplement will be selected for use in the vinasse culture medium. The objective of this section is to develop a series of tests aimed at determining the optimal levels of parameters that affect bacterial growth and urease activity in the selected culture medium. In this study, an L9 orthogonal array of TDOE was employed to design the experiments.

To explain more, to optimize test design and evaluate input factors, a suitable orthogonal array is chosen based on identified factors and their appropriate levels. Full factorial design (FFD), considering all parameter combinations, can be time-consuming and costly due to the high experiment count. Fractional factorial experiments (FFEs), developed by Taguchi (1987), reduce the number of required tests while employing standard orthogonal arrays introduced by Fisher in the 1920s^84,85^. In fact, TDOE uses a subset of parameter combinations in L9 orthogonal array to optimize design and assess input factor effects efficiently. In this study, an L9 orthogonal array of TDOE was employed to design the experiments, ,as previous studies showed that L9 orthogonal array can provide appropriate level of precision^86^.

Therefore, in this study, three affecting parameters of vinasse concentration (V) in v/v%, incubation time, and selected supplement concentration (w/v%) are considered as the influencing factors, each one at three levels.

To determine the optimum level of each parameter, the analysis of means (ANOM) was done on the signal-to-noise ratios (S/Ns) of the test results. The values of S/Ns were determined for each influencing factor at different levels as follows, considering larger-the-better criteria (Eq. 5):

5 $$S/N=-10{\text{log}}\left(\frac{\sum_{i=1}^{r}(\frac{1}{{X}_{i}}{)}^{2}}{r}\right),$$

where *r* and ${X}_{i}$ are the number of repetitions and the test results, respectively.

The mean of the S/N ratios for factor f at level l (${M}_{{\text{S}}/N}$) can be computed using the following formula (Eq. 6):

6 $${({M}_{{\text{S}}/N})}_{Level=l, Factor=f}=\frac{1}{{n}_{fl}}\sum_{i=1}^{{n}_{fl}}[(\frac{S}{N}{)}_{l,f}{]}_{i},$$

where ${n}_{fl}$ is the number of repetitions of factor f in level l.

In fact, based on the ANOM analysis outcomes and adherence to the fundamental criteria, one can determine the optimal level for each factor.

Moreover, the research involved performing an analysis of variance (ANOVA) to assess the extent of involvement, or degree of participation (DOP), attributed to each of the influencing factors. This statistical technique allowed us to quantify how much each factor contributed to the observed variations in the data.

To delve deeper into our findings, we examined the results obtained from the Analysis of Means (ANOM) analyses. This involved a direct comparison of the factor with the highest DOP as determined by the ANOVA analysis with the influencing parameter that exhibited the greatest significance in the ANOM analysis. This comparison allowed us to understand the interplay between the factors and their impact on the observed effects, shedding light on which factors played a more prominent role in the analyzed data (for further discussion about TDOE, ANOVA, and ANOM refer to Mortazavi Bak, Noorbakhsh, Halabian, Rowshanzamir and Hashemolhosseini^87^).

## Results and discussions

### Appropriate enriched Vinasse culture medium

In the context of introducing a cost-effective culture medium for cultivating the *S. pasteurii* bacterium with the aim of reducing the costs associated with the MICP biotechnology method, "enriched vinasse" refers to vinasse, a byproduct of ethanol production, that has been fortified to provide an optimal nutritional culture medium conducive to the growth and proliferation of the *S. pasteurii* bacterium. This enrichment process encompasses the addition of essential supplements, pH adjustment, and sterilization to enhance the suitability of vinasse as a culture medium for *S. pasteurii*, ensuring their efficient growth and urease activity in MICP biotechnological applications, such as improving soil strength parameters.

**Figure 4:**
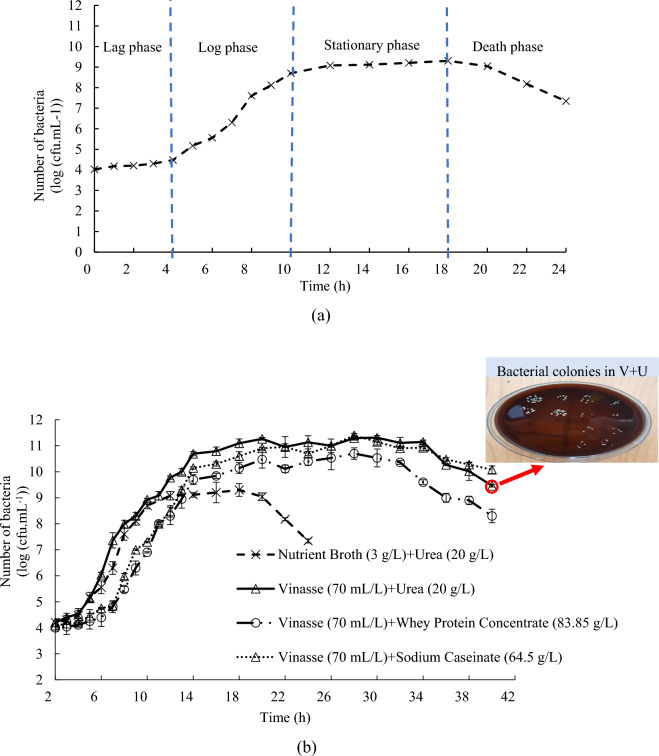
(**a**) Growth phases of *S. pasteurii* bacterium in NB + U culture medium, (**b**) The *S*.* pasteurii* bacterium growth curves on protein-rich vinasse and NB + U culture media.

In this section, the behavior of *S. pasteurii* bacterium incubated within the considered culture media is assessed based on their growth curves. Figure 4b presents growth curves of *S. pasteurii* for different culture media (i.e. V + U, V + NaCAS, and V + WPC). The experiments were conducted with three replications, under the same conditions, and the growth curves were plotted based on the mean values of the data. The growth curves were drawn until the bacteria growth process entered the death phase in each culture medium, reaching 24 h for NB + U culture medium and 40 h for the various enriched vinasse culture media. In fact, the growth curves exhibit all four possible phases, including the lag (delay), log (logarithmic), stationary, and death phases (Fig. 4a).

As seen in Fig. 4a, for the conventional culture medium (NB + U), the *S. pasteurii* bacterium enters the stationary phase approximately 10 h after the beginning of growth, with a maximum number of grown bacteria of 10^9.3^ per milliliter. In contrast, when using the proposed culture media, the bacterium achieved higher maximum reproductive rates, but entered the stationary phase over a longer period (around 14 h). On the other hand, the maximum number of bacteria significantly increased to 10^9.7^–10^11.4^ (Fig. 4b).

The enriched vinasse culture media have shown a significant increase in the maximum bacterial multiplication, exceeding 2 logarithmic units when compared to NB + U culture medium under identical conditions. In contrast, previous studies did not observe a substantial boost in *S. pasteurii* bacterial growth when exploring alternative waste materials, such as corn steep liquor, lactose mother liquor, and yeast extract, previously introduced as culture media for *S. pasteurii*, in comparison to NB + U culture medium^39,42,44,45,88^. Additionally, the stationary phase in the proposed culture media lasts over 20 h, which is significantly longer than the conventional culture medium’s stationary phase of 10 h. The longer stationary phase is particularly important for practical applications of the MICP method since it allows more time to introduce bacteria into the soil.

The higher number of bacteria in the culture medium can lead to more effective soil treatment due to the increased production of urease enzyme within the soil. Based on the growth curves, it appears that the V + U culture medium is the most effective one for growing *S. pasteurii* bacterium among other considered culture media. Nevertheless, it is worth noting that the differences in the growth curves between the various media are relatively small and the urease activity of the bacteria may play a crucial role to adopt the most appropriate culture medium for the MICP procedure. Therefore, additional tests were conducted to examine the urease activity of *S. pasteurii* bacterium grown within the different culture media.

As discussed previously, Urease activity was determined for *S. pasteurii* bacterium in the different culture media including N + U, V + NaCAS, V + WPC, and V + U, by measuring the amount of ammonia released from hydrolyzed urea using the Nessler method (at 425 nm, 25 °C, pH 7). To identify the most appropriate culture medium, the urease activity of *S. pasteurii* bacterium was measured in different media in the middle of their stationary phase, i.e. 14 h for conventional culture medium and 24 h for the proposed culture media (Table 1).

**Table 1:** The result of urease activity tests for *S. pasteurii* bacterium in various culture media.

Culture media	NB + U	V + U	V + NaCAS	V + WPC
Urease activity (U/min)	216	447*	386	312

*Highest urease activity.

The results of the urease activity tests show that the urease activity of *S. pasteurii* bacterium in the new culture media is significantly greater than NB + U medium while the highest value devotes to the V + U culture medium. Additionally, the use of enriched vinasse culture media has been shown to enhance the urease activity of *S. pasteurii* bacterium, i.e. 447 U/min, to a greater extent than waste materials such as corn steep liquor (less than 300 U/min), lactose mother liquor (less than 250 U/min), yeast extract (less than 270 U/min) which were previously introduced as culture media for the *S.pasteurii* bacterium^39,42,88^.

For economic assessment purpose, the costs of the considered culture media were estimated by comparing the prices of all elements used in the proposed media in terms of the average industrial prices in Iran. The estimated costs are presented in Table 2.

**Table 2:** Estimated cost of different culture media considered for MICP treatment.

Culture media	NB + U	V + U	V + NaCAS	V + WPC
Cost*/L (US$)	0.455	0.005	0.315	0.551
Relative cost in comparison to NB + U	1	0.011	0.692	1.210

*Approximate industrial grade price (current market).

In Table 2, the V + U culture medium emerges as the most economically efficient option, costing just $0.005 per liter. This cost-effectiveness is especially pronounced when compared to the conventional NB + U culture medium, which costs $0.455 per liter. The significant cost difference can be attributed to the abundance and cost-free nature of vinasse as an abundant industrial-agricultural waste, a pivotal ingredient in the V + U culture medium. In contrast, traditional culture media rely on commercial components that contribute significantly to their overall cost. Furthermore, when considering supplements like NaCAS and WPC, the cost savings with the V + U culture medium become even more apparent. This reduction in expenditure makes the MICP technique economically viable and broadly accessible.

This study found that the combination of vinasse and urea provides suitable conditions for the growth and production of the urease enzyme. The results in fact showed that urea was the most effective supplement for the growth of *S. pasteurii* bacterium in the culture medium containing vinasse. Previous research has also demonstrated that urea is the suitable supplement for the growth of the bacterium within other culture media^89,90^.

In summary, the use of urea-enriched vinasse (V + U) culture medium can significantly improve the efficiency of the MICP method by increasing both the urease activity and number of bacteria. Moreover, it is the most cost-effective culture medium among the considered alternatives. Hence, V + U culture medium is suggested as the best culture medium alternative and further tests are conducted to optimize this medium for soil improvement purposes.

### Optimum culture medium

As highlighted in the preceding section, the utilization of vinasse as a fundamental constituent of bacterial culture medium in the MICP brings about an upsurge in the number of bacteria and urease activity, while simultaneously rendering the MICP process cost-effective. Optimizing the proportion of vinasse, which contains moderate levels of nitrogen, as well as other organic and inorganic elements, within the proposed culture medium is a crucial aspect of this study. To ascertain the ideal proportion of vinasse and urea in the culture medium, the study utilized TDOE, ANOVA, and ANOM to evaluate the number of bacteria (i.e. log (cfu/mL)) and the urease activity of *S. pasteurii* (i.e. UA (U/min)). The study employed the L9 array to design the experiments, and based on the initial trials, it was found that the most suitable values for Vinasse Concentration, Incubation Time, and Urea Concentration lie between 4–10%, 10–30 h, and 1.5–2.5%, respectively. These values were determined to yield an optimal number of bacteria and urease activity.

Table 3 displays the logarithmic values of bacterial number and urease activity for the T1 to T9 experiments. Previous research has demonstrated that the TDOE method can predict outcomes with a satisfactory level of accuracy^20,86,87,91^. Table 3 also includes the predictions of the TDOE method, which were calculated using the Minitab software package^92^ for full factorial test conditions, as well as the S/N ratios obtained using Eq. (1).

**Table 3:** Comparison between the experimental results and TDOE predictions.

	Factor level			log (cfu/mL)			UA (U/min)		
	V (%)	Time (h)	U (%)	Test	TDOE	S/N	Test	TDOE	S/N
T_1_	4	10	1.5	6.81	6.63	16.10	327.86	325.54	50.73
				6.03			362.42		
T_2_	4	20	2	9.29	8.57	19.89	323.59	308.96	50.47
				10.59			344.71		
T_3_	4	30	2.5	5.81	7.10	15.47	218.98	260.66	46.68
				6.07			212.76		
T_4_	7	10	2	6.24	7.74	16.33	247.98	299.97	48.13
				6.92			262.38		
T_5_	7	20	2.5	9.94	10.29	20.07	384.57	359.75	51.58
				10.22			374.13		
T_6_	7	30	1.5	11.56	10.35	21.38	431.65	401.61	52.60
				11.88			421.95		
T_7_	10	10	2.5	9.46	8.31	19.71	421.57	393.11	52.43
				9.90			415.03		
T_8_	10	20	1.5	11.10	12.41	21.02	513.45	543.03	53.94
				11.40			483.03		
T_9_	10	30	2	9.97	10.33	20.10	441.08	418.38	52.83
				10.27			434.88		
T_10_	4	10	2	–	5.71	–	–	224.58	–
T_11_	4	10	2.5	–	5.40	–	–	239.99	–
T_12_	4	20	1.5	–	9.49	–	–	389.91	–
T_13_	4	20	2.5	–	8.26	–	–	304.36	–
T_14_	4	30	1.5	–	8.33	–	–	346.22	–
T_15_	4	30	2	–	7.41	–	–	265.26	–
T_16_	7	10	1.5	–	8.65	–	–	380.93	–
T_17_	7	10	2.5	–	7.42	–	–	295.38	–
T_18_	7	20	1.5	–	11.52	–	–	445.30	–
T_19_	7	20	2	–	10.60	–	–	364.35	–
T_20_	7	30	2	–	9.44	–	–	320.65	–
T_21_	7	30	2.5	–	9.12	–	–	316.05	–
T_22_	10	10	1.5	–	9.54	–	–	478.66	–
T_23_	10	10	2	–	8.63	–	–	397.70	–
T_24_	10	20	2	–	11.49	–	–	462.08	–
T_25_	10	20	2.5	–	11.18	–	–	457.48	–
T_26_	10	30	1.5	–	11.24	–	–	499.34	–
T_27_	10	30	2.5	–	10.04	–	–	413.78	–

Table 4 summarizes the results of ANOM as per Table 3 test results. According to Table 4 and considering the fact that larger values of number of bacteria and urease activity can cause better outcomes for microbial soil treatment purposes, the optimal levels for Vinasse concentration (V), incubation Time, and Urea concentration (U) are the third, second, and first levels, respectively. The ANOM analysis indicates that higher Vinasse concentration, as the most influential parameter, leads to increased levels of urease activity and number of bacteria, with 1.5% of urea being the most effective concentration for optimal culture medium. The optimal percentage of the urea is lower in the proposed culture medium, i.e. 1.5% compared to 2% considered in previous studies for *S. pasteurii* bacterium^34,39,42,65,88^. This variation can be attributed to the nitrogen content present in the vinasse substance. The cost of waste culture media is contingent on the costs of supplements needed for bacterial growth. The proposed culture medium utilized in this study necessitates smaller quantities of urea as a supplement, rendering it a more cost-effective option.

**Table 4:** Summary of ANOM results.

Effecting parameters	Mean S/N ratio			Significance of parameter
	Level 1	Level 2	Level 3	Max–min
log (cfu/mL)				
V (%)	17.16	19.26	20.28*	3.12*
Time (h)	17.38	20.33*	18.98	1.60
U (%)	19.50*	18.77	18.42	1.08
UA (U/min)				
V (%)	49.29	50.77	53.07*	3.77*
Time (h)	50.43	51.99*	50.70	1.57
U (%)	52.42*	50.47	50.23	1.95

*The most affecting parameter.

To summarize, the choice of 10% Vinasse concentration, 1.5% Urea concentration, and a 20 h incubation time as optimal parameters for effective microbial soil treatment is rooted in the observation that higher Vinasse concentrations lead to increased urease activity and bacterial counts. The use of 1.5% of urea is particularly effective and cost-efficient due to reduced urea supplement requirements, thanks to the inherent nitrogen content in vinasse. Furthermore, the statistical analysis and findings presented in Fig. 4, which show that at the 20-h growth time, *S. pasteurii* bacteria enter the early stages of their stationary phase in the V + U culture medium, support 20 h incubation time, as it reveals that *S. pasteurii* experiences greater proliferation at 20 h of incubation compared to 10 h, with the bacteria being younger than those at 30 h, contributing to improved growth and enhanced urease activity.

The findings from both the ANOVA and ANOM analyses exhibit congruency, indicating that the parameter V exerts the most pronounced influence, with both the highest level of significance (3.77) and the highest degree of participation (55.25%). In contrast, Time emerges as the least influential parameter, consistently aligning with the results obtained from the ANOM analyses.

### Effects on soil microbial stabilization

To investigate the impact of using the optimal culture medium on bio-treated soil, a series of direct shear tests were conducted on loose and bio-treated sand specimens. The tests were performed using both the fresh optimal culture medium (V(100 mL/L) + U(15 g/mL)) and the conventional culture medium (NB (3 g/L) + U (20 g/L)). The data obtained from the direct shear tests (Table 5) indicate a significant increase in the shear strength parameters when using the proposed culture medium. Compared to the untreated soil, optimum culture medium exhibited an increase of 7.6° in the friction angle and 219.5 kPa in cohesion. On the other hand, the conventional culture medium resulted in an increase of 5.1° in the friction angle and 198.8 kPa in cohesion. This increase in shear strength parameters can be attributed to the higher number of bacteria and increased urease activity of the optimal culture medium. Additionally, vinasse, which is a calcium-containing biopolymer, also has a positive impact on soil stabilization as a soil stabilizer. To sum up, the use of the newly introduced culture medium for the MICP method will result in a noteworthy enhancement in the shear strength of soil samples. This improvement can be mainly attributed to the augmented cohesion between soil particles, as previously demonstrated in studies^16,93,94^.

**Table 5:** Strength parameters of soil and MICP-treated soil using the proposed culture media and control one.

Culture media	None untreated	NB(3 g/L) + U(20 g/L)	V(100 mL/L) + U(15 g/mL)
Friction angle ($^\circ$)	31.0	36.1	38.6
Cohesion (kPa)	0	198.8	219.5

**Figure 5:**
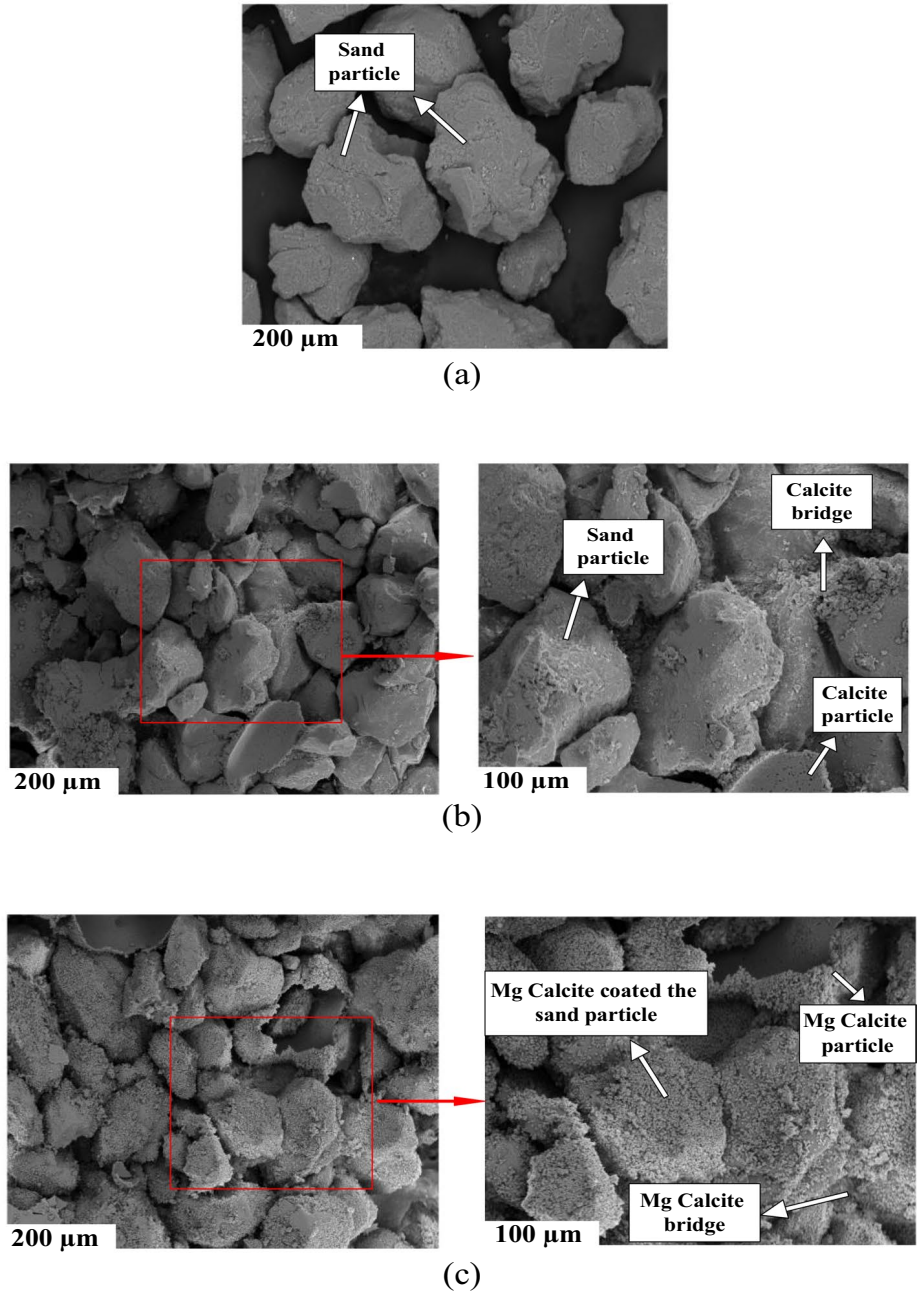
SEM images; (**a**) untreated sand & (**b**) treated sand by *S. pasteurii* grown in NB (3 g/L) + U (20 g/L) in the 200 and 100 µm resolutions, (**c**) treated sand by *S. pasteurii* grown within V (100 mL/L) + U (15 g/L)) in the 200 and 100 µm resolutions.

SEM images of soil samples were obtained to assess the quality of microbial calcite precipitation within soil pores and on the surface of soil particles following the bio-treatment. The SEM images of both untreated and treated soil samples are presented in Fig. 5.

Upon examination of Fig. 5, it is evident that the use of new culture medium results in the thorough coating of sand particles, and the formation of calcite bridges between these particles is also noticeable. These bridges enhance the cohesion of the bio-treated soil medium. It's important to highlight that XRD analyses were performed to determine the crystal type formed in soils treated with MICP. This will be further elaborated in the following discussion.

**Figure 6:**
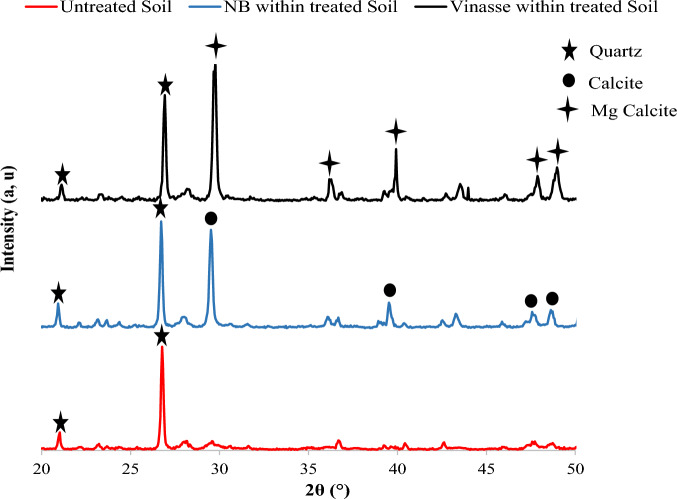
The X-ray diffraction (XRD) pattern for soil samples, untreated and treated was obtained using the NB + U and optimum V + U culture media.

Additionally, different types of calcium carbonate ions (e.g. calcite, aragonite, and vaterite) can be produced in an MICP process, but amongst these carbonates, calcite is the most stable one, hence is preferred when an MICP treatment is adopted^24,47,95^. To identify the type of calcium carbonates created in the MICP-treated soil samples of this study, the XRD technique was adopted. Figure 5 presents the XRD analysis of loose soil, and bio-treated samples by conventional and the proposed culture media. The results show that the formed crystals in the sample by the conventional culture medium were mainly of calcite type, while these crystals were magnesium calcite crystals (i.e. Mg_0.1_Ca_0.9_CO_3_) in using the optimum V + U culture medium. The reason for the presence of magnesium in the calcium carbonate crystals is the result of using the new proposed culture medium and the present of magnesium elements in the vinasse substance. The maximum intensity of the crystals in both MICP treated samples were at the 2θ between 29.6° and 29.8° which is in agreement with several previous studies in this regard^24,64,96,97^. Figure 6 shows that the characteristic diffraction peak of quarts was also detected, as expected considering that the soil used in this study was a quartz sand.

It is worth noting that magnesium calcite crystals have a more complex and interlocking structure than pure calcite crystals, which gives them better soil stabilization and erosion resistance. Previous research has shown that adding low concentrations of Mg^2+^ can improve the mechanical properties of sand specimens and achieve a better reinforcement effect when using MICP to consolidate the soil^98^.

Therefore, vinasse can be considered a sustainable and cost-effective source of magnesium for the production of magnesium calcite crystals in MICP and the presence of magnesium in calcium carbonate crystals, particularly when derived from vinasse in MICP treatment, consider as a promising approach for enhancing soil stabilization with improved durability, sustainability, and cost-effectiveness.

Given the outcomes of this study, practical applications like soil stabilization in construction, road construction and desert environments can take advantage of the V + U culture medium's affordability and accessibility. With a cost reduction of up to 91 times compared to the traditional NB + U medium, MICP becomes a significantly more practical and sustainable solution for a wide range of industries and global projects. This substantial cost reduction is primarily due to the use of vinasse, a readily available industrial-agricultural waste product, as a primary component in the V + U medium, as opposed to the costly commercial ingredients found in conventional media.

## Conclusions

The study aims to enhance the cost-effectiveness of the MICP method for soil improvement. Vinasse (V) was investigated as a primary component of the culture medium, providing a cost-effective alternative to the traditional NB culture medium. Tests were conducted to assess *S. pasteurii* bacterial growth and urease activity using three Vinasse-based culture media with varying supplements like with urea (U), sodium caseinate (NaCAS), and whey protein concentrate (WPC). Among these, the V + U culture medium emerged as the most effective alternative to the conventional culture. The discovery of this novel culture medium’s ability to potentially cut bacterial culturing costs by up to 91 times compared to the conventional medium underscores the practical significance of this research. This is particularly significant because a significant barrier to the field-scale implementation of MICP method has been the prohibitively high cost of nutrient culture media required for cultivating the necessary bacteria. In this study, the enriched vinasse serves as a sustainable and cost-effective nutrient source for producing urease enzymes and magnesium calcite crystals in MICP. These crystals improve soil stabilization significantly, with a 219.5 kPa increase in cohesion and a 7.6° friction angle improvement compared to untreated soil. This approach has practical applications beyond the lab, especially in large-scale projects. Additionally, recycling vinasse into a valuable product aligns with the circular economy concept. In summary, this research advances MICP soil improvement with a cost-effective, sustainable solution, demonstrating the potential to revolutionize large-scale soil improvement practices for a more efficient and eco-friendly future.

Future studies can explore the behavior of other bacteria in the proposed culture medium and investigate the use of other waste materials as the basic ingredient for developing low-cost culture media or introduce other studied supplements for vinasse like including low-grade or technical-grade yeast extract, whey, and lactose mother liquor.

In conclusion, we recommend the V + U culture medium for MICP large-scale applications due to its benefits. Future research should explore its effectiveness in various applications beyond soil improvement, such as interface soil-steel or soil-concrete interactions, stone joint filling, and other potential uses to broaden its practical applicability and impact.

### Supplementary Information

Supplementary Figure S1.

## Data Availability

The datasets generated during and/or analysed during the current study are available from the corresponding author on reasonable request.
