# Supplementary material for: Soil microbial improvement using enriched vinasse as a new abundant waste
Source: Sci Rep. 2023 Dec 14;13:22279. doi: 10.1038/s41598-023-49401-w (PMC10721901; doi:10.1038/s41598-023-49401-w)
Supplement: Supplementary file 1 — Supplementary Figure S1. [file 41598_2023_49401_MOESM1_ESM.docx]

Supplementary Materials

**Soil microbial improvement using enriched vinasse as a new abundant waste**

**Tahereh Kariminia^a^, Mohammad A. Rowshanzamir^a^, S. Mahdi Abtahi^[[1]](#footnote-1)^ ^a^, Sabihe** **Soleimanian-Zad^b^,** **Hamid Mortazavi Bak^c^,** **Alireza Baghbanan^d^**

S1: Schematic diagram of Miles-Misra method

1. Corresponding Author.

   Email Address: mabtahi@iut.ac.ir [↑](#footnote-ref-1)
